# Pavement crack identification method based on IOtsu-Dd algorithm

**DOI:** 10.1371/journal.pone.0322662

**Published:** 2025-05-14

**Authors:** Yang Yang, Lin Wang, Qinghua Xiong

**Affiliations:** 1 School of Road and Bridge Engineering, Guangxi Transport Vocational and Technical College, Nanning, China; 2 Hebei Expressway Hangang Expressway Co., Ltd., Cangzhou, China; Beijing Institute of Technology, CHINA

## Abstract

Rapid identification of highway cracks is greatly significant for highway maintenance. In recent years, the use of unmanned aerial vehicles to collect images of road cracks for automatic recognition has become a topic of concern for many researchers. Based on this, to raise the accuracy and efficiency of crack recognition, a road crack recognition method based on unmanned aerial vehicle images and improved Otsu method is developed. Firstly, certain processing techniques are applied to the images captured by the unmanned aerial vehicle, such as grayscale and equalization, to reduce computational complexity and facilitate subsequent identification of image cracks. Subsequently, to improve recognition accuracy, the image is segmented and the Otsu method is introduced and improved. Finally, a pavement crack recognition model is constructed using damage density, achieving the extraction and recognition of pavement crack features from images. The experiment findings show that the raised recognition model has an average accuracy of 98.2%, a recall rate of 0.75, and an F1 score of 0.85 in crack recognition of unmanned aerial vehicle captured images. This denotes that the raised recognition model has strong effectiveness and high recognition accuracy, and the method can effectively recognize road cracks based on unmanned aerial vehicle images.

## 1. Introduction

With the rapid development of highway construction, many industries are increasingly inseparable from a well-developed highway transportation network [1]. However, with the passage of time and the increase of vehicles, the road surface of highways has experienced varying degrees of damage. The identification technology of road cracks (RCs) is becoming increasingly important for accurate maintenance of the road surface. Meanwhile, with the widespread use of unmanned aerial vehicle (UAV) technology, how to combine the high efficiency of UAV technology with crack recognition technology has become an important issue for researchers. Adding UAV technology to RC identification has many benefits. UAVs can quickly cover large areas of road surfaces, reducing the waste of human resources and time costs [2]. Compared to manual inspection, UAVs can complete crack detection tasks more quickly. At the same time, UAVs can capture and record cracks on the road surface in real time, avoiding the problems of subjective misjudgment and missed detection by humans [3]. The use of UAVs for crack detection can promptly detect and record RCs, providing timely and accurate information for relevant departments and facilitating repair and maintenance. The traditional crack detection method requires a lot of manpower and time, and it is costly to conduct comprehensive inspection on a large area of highway pavement. UAVs for crack inspection can reduce operating costs, improve detection efficiency and accuracy, thereby reducing maintenance and repair costs for relevant departments [4]. In addition, during crack inspection by drones, the target cracks are relatively small compared to the background, and the number of pixels occupied by crack features in the image is very limited. Traditional image processing algorithms, such as edge detection, are prone to noise interference when processing small features, leading to a decrease in recognition accuracy. Small cracks are often confused with other details on the road surface, such as textures, shadows, etc., increasing the risk of misclassification [5,6]. At present, although modern image processing techniques such as deep learning can handle high-resolution and low resolution images, ultimately alleviating this problem, there are still limitations. One is that deep learning relies on a large number of samples, and deep learning models typically require a large number of labeled samples for training. The lack of small crack samples can limit their effectiveness, especially in areas of data scarcity. Secondly, deep learning methods have relatively weak decision transparency and model interpretability, which is crucial for maintaining public infrastructure. Therefore, to raise the accuracy of RC recognition and cut down costs, an RC recognition method with UAV images and improved Otsu (Iotsu) method is developed. The method processes the images collected by the UAV and segments them using the Iotsu algorithm. Finally, an RC recognition model (Iotsu-Dd) is constructed using damage density (Dd) to extract and recognize RC features.

The innovation of the research lies in the balance between the accuracy and efficiency of image segmentation. By using the weighting factor of pixel distribution to adjust the selection of threshold, the segmentation accuracy of the algorithm is improved when the crack target is small or the background is complex. At the same time, the DD feature extraction method is used to improve the accuracy of the model without significantly increasing the complexity of the model through more effective feature selection. At the same time, the accuracy of pavement crack recognition is improved by image preprocessing, Otsu algorithm improvement and feature extraction combined with the geometric characteristics of cracks. In addition, the running time is effectively reduced by image dimension reduction and optimization calculation process. Through image processing methods such as grayscale, histogram equalization and gradient inverse weighted filtering, the dimension of image data is reduced and the computational complexity is reduced. By replacing the standard pixel distribution probability calculation and using weighted histogram, the algorithm can find the appropriate threshold for segmentation more quickly when dealing with complex background. This optimization reduces the computational complexity, thus reducing the running time.

The research is composed of four parts. Part 1 provides an overview of RC recognition methods based on UAV images and Iotsu algorithm. Part 2 processed the UAV images to some extent and improved the IS algorithm Otsu, proposing the Iotsu-Dd recognition model. Part 3 conducts experiment verification on the raised method. Part 4 summarizes the experiment outcomes and proposes future prospects.

## 2. Related works

The automatic recognition of RCs is of great significance for highway maintenance, and good IS algorithms can greatly improve the accuracy of crack recognition models. Yang P et al. developed an enhanced threshold bias adjustment strategy to address the issue that the threshold generated by the Otsu algorithm is frequently closer to the category with the greater intra-group variance. The outcomes denoted that improving the algorithm performance significantly reduced the time consumption of the algorithm [7]. In the paper, the study improves the applicability of Otsu algorithm in situations with large inter class variance by enhancing threshold bias adjustment, but still relies on global histogram statistics, which may lead to misjudgment of crack areas in complex backgrounds or low contrast situations. Chen L et al. developed a new adaptive fractional order genetic particle swarm optimization algorithm (FOGPSO) to improve the Otsu algorithm. The experimental results indicated that FOGPSO outperformed other methods in both qualitative and quantitative aspects [8]. The paper combines genetic algorithm and particle swarm optimization algorithm, making Otsu algorithm more adaptive and improving segmentation performance. However, its computational complexity is relatively high, and compared to the standard Otsu algorithm, the computation time increases, making it unsuitable for large-scale real-time detection. To enhance the real-time efficacy of IS, Huang C et al. integrated the fruit fly optimization algorithm (FOA) into the Otsu segmentation approach, thereby developing an FOA-OTSU algorithm. Simulation showed that the new algorithm converged faster and consumed less time [9]. The new algorithm combined with fruit fly optimization algorithm improves the stability of Otsu threshold selection, enables faster convergence, and enhances computational efficiency. However, the stability of the global optimal solution needs to be improved, and local optimal problems may arise. To address the deficiencies of one-dimensional and two-dimensional Otsu thresholding techniques, Bhandari AK et al. developed a three-dimensional Otsu method. The developed method focused on preserving edge details by calculating the three-dimensional Otsu along the fusion phenomenon. The results indicated that the developed method produced more results [10]. The study uses the three-dimensional Otsu method to perform finer grained segmentation in spatial and color information, improving edge preservation ability. However, its computational complexity has significantly increased, and compared to traditional Otsu methods, the computation time has increased, which may affect real-time performance. To address the issue of building change detection, dos Santos R C et al. introduced an automatic detection method for building changes ground on Otsu algorithm and median flatness attribute calculated from eigenvalues. The outcomes denoted that the new method exhibited approximately 99% and 76% completeness and correctness [11]. The study improves the accuracy of building change detection by combining Otsu method and median flatness attribute, and is suitable for crack detection. But it is mainly used for building change detection, and direct application to road crack detection may require further optimization and adjustment.

Hou Y et al. developed an intelligent crack pattern recognition method grounded on limited field of view images to solve the problem of automatic recognition of RCs. At the same time, model selection and hyperparameter tuning were performed for CNN. The results indicated that the method could effectively identify crack images in asphalt road [12]. The study combines deep learning (CNN) for crack recognition and has good generalization ability, which can adapt to different types of crack shapes. But it requires a larger dataset for training, and performance may be limited for small sample datasets. Yang Q et al. developed a vibration-based method for identifying transverse cracks in asphalt road to address the issue of RC detection. The vibration signals of the vehicle in motion were measured and analyzed in a preliminary manner to ascertain their nature in the transverse crack section and adjacent uncracked sections. This was conducted in the time domain. The outcomes denoted that the method could quickly identify RCs [13]. The study uses vehicle vibration signals for crack detection, reducing dependence on image quality and lighting conditions and improving adaptability. But it is only applicable to specific types of cracks, and the detection effect is poor for longitudinal cracks or subtle cracks. To address the issue of intelligent recognition of RCs, Ma D et al. used a fully convolutional neural network based on ResNet-101 to build an intelligent recognition model for RC areas. The substitution of a convolutional layer for a fully connected one enabled complete convolutional operation and markedly enhanced computational efficiency. The outcomes denoted that the model had broad application prospects in practical engineering problems [14]. The study uses ResNet-101 as the backbone network to improve the accuracy and robustness of crack area recognition. But it requires a large amount of annotated data for training, and insufficient data may lead to a decrease in model performance. Tran TS et al. developed a two-step continuous automatic process for detecting, identifying, and recognizing cracks in asphalt road management systems, aimed at identifying their severity. The experimental results indicated that the model could be used for detecting and identifying RCs [15]. The study improves the processing efficiency of the system by dividing crack detection and severity identification into two independent steps. This structural design has good flexibility and can be optimized according to actual needs. However, this method relies on high-quality image preprocessing in the stage of identifying crack severity. Li B L et al. developed and described a new model for crack recognition in asphalt road, which is based on the fusion of grid-based recognition and box-based detection in You Only Look Once version 5 (YOLO v5). The outcomes denoted that this method effectively achieved automatic identification of cracks in asphalt road [16]. The study combines grid recognition and box detection to improve the accuracy of crack recognition. But the detection effect for smaller cracks may be poor and susceptible to background interference. Fan L et al. developed a new dataset of road shadows and cracks to address the interference problem of road shadows in the process of RC detection, and designed a crack detection method for two-step shadow removal. The method demonstrated enhanced algorithmic performance through the initial removal of shadows, followed by their subsequent detection. The results of the experiment revealed that this method is more effective than other methods [17]. The study, due to its shadow removal strategy, can handle complex lighting conditions and shadow distribution problems, and has strong adaptability, especially suitable for outdoor road scenes with variable shadows. However, it requires high quality of image data, and low-quality or excessively noisy images may affect the effectiveness of shadow removal, leading to a decrease in subsequent crack detection results. Ha J et al. suggested a system for automatic detection and classification of RCs using SqueezeNet, and other models. Meanwhile, a separately trained U-net was used to perform linear and regional crack segmentation on the input image. The outcomes denoted that the developed detection system had good recognition performance [18]. The U-Net model used for image segmentation, as well as the combination of SqueezeNet and MobileNet SSD, can effectively detect and segment cracks in complex backgrounds. However, this method relies on a large amount of training data and computing resources, which limits its detection performance in practical application scenarios, especially when data is scarce or annotation costs are high. The method proposed by the research institute can achieve more reasonable recognition results without the need for a large dataset, and has more practical application value. Li B et al. suggested a novel method for automatic classification of 3D road surface images using deep cellular neural networks to address the classification problem of road surface cracks. Four supervised cellular neural networks with different receptive fields were successfully trained. The experimental results indicated that all developed cellular neural networks could perform high-precision classification [19]. Compared with the method proposed by the research institute, this method may improve the classification accuracy in complex situations. However, due to its complex structure, its computational response speed and real-time processing capability are insufficient compared to the proposed method. In road crack identification, computational efficiency and real-time processing capability are of great significance for road maintenance.

In summary, numerous scholars have conducted extensive research on RC recognition methods based on drones. However, these studies have not achieved a good balance between detection accuracy and real-time computational efficiency. The proposed method optimizes the processing and segmentation of drone images, ensuring both detection accuracy and real-time image processing capabilities, providing an effective recognition technology for automatic identification of road cracks based on drone images.

## 3. Road crack recognition based on UAV images and Iotsu algorithm

To solve the problem of large-scale road maintenance, it is necessary to accurately and efficiently identify RCs. Based on this, an RC recognition method based on UAV images and Iotsu algorithm is developed. The method first preprocesses the RC images collected by the UAV, aiming to raise the accuracy of crack recognition in the next step of the image. Subsequently, the Otsu algorithm is introduced and improved, using the improved algorithm to segment RC images. Finally, the Iotsu-Dd model is constructed using Dd to extract and recognize the features of road surface cracks in images.

### 3.1. Road crack image processing based on UAV image

**Fig 1:**
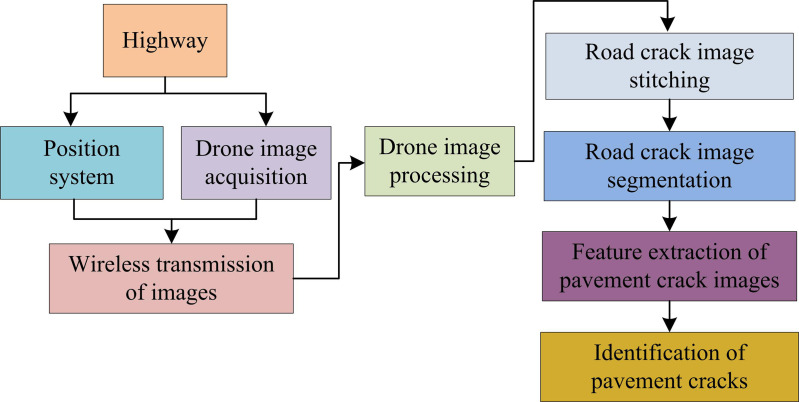
Process diagram for identifying road cracks.

To accurately and efficiently identify RCs, UAVs are utilized to capture images of the road surface, and the captured images are transmitted wirelessly to the receiver. After receiving, the receiver sends it to the processing system for image preprocessing. The flowchart for identifying RCs is shown in Fig 1.

**Fig 2:**
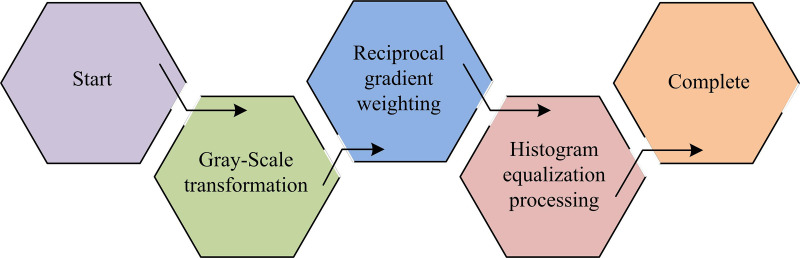
Schematic diagram of UAV road crack image enhancement.

In Fig 1, the road is captured through UAV aerial photography, and the resulting video images are wirelessly transmitted to the receiver. At the same time, this process uses a positioning system to perform real-time positioning and tracking of the UAV’s aerial route. The receiver transmits the image data to the processing system for image preprocessing. Firstly, to solve the problem of incomplete images of large cracks caused by the field of view captured by UAVs, image stitching technology is used for stitching to obtain complete crack information. Subsequently, IS techniques are used to separate RCs from the background image. The characteristics of the segmented RC images are then extracted and identified, and the cracks are evaluated. To raise the accuracy of identifying RCs, it needs to strengthen the images captured by UAVs. The image enhancement method is shown in Fig 2.

In Fig 2, the enhancement of the UAV RC image is first processed with grayscale to reduce the problem of excessive computation caused by image color. Subsequently, to address the issue of uneven grayscale distribution in road surface images, histogram equalization was employed for processing. Finally, to reduce image noise, gradient inverse weighting is used for filtering. The grayscale range is 0–255, the number of bins for histogram calculation is set to 256, and the size of the gradient inverse weighted filtering window is 3 × 3. To address the issue that the original images captured by UAVs are color images that are not conducive to subsequent calculations, grayscale processing is applied. Firstly, the pixel values of the pixels in the image are weighted and averaged, as shown in Equation 1.


$$g_{ij}=w_{1}R_{ij}+w_{2}G_{ij}+w_{3}B_{ij}$$ (1)

In Equation 1, $g_{ij}$ means the grayscale value of pixel (i,j). $w_{1}$, $w_{2}$, and $w_{3}$ represent the weights of pixel value components, respectively. $R_{ij}$, $G_{ij}$, and $B_{ij}$ respectively represent different components at the pixel. Among them, the weights of the components have the relationship shown in Equation 2.


$$w_{1}+w_{2}+w_{3}=1$$ (2)

Grayscale processing of images can help reduce computational complexity, but after grayscale processing, the grayscale distribution of road images may be uneven, leading to issues with image highlights and unclear road background and defect boundaries [20]. According to this, histogram equalization is utilized to further process the image. Histogram equalization is the process of transforming the original image through a function to regulate its grayscale distribution, resulting in a new image with a reasonable histogram distribution. This is used to adjust the brightness of the image and enhance the contrast of images with smaller dynamic ranges [21]. The fundamental concept underlying histogram equalization is the widening of grayscale levels with a greater proportion within the image, and the compression of grayscale levels with a smaller proportion. This process serves to enhance the uniformity of the histogram distribution within the image, expand the dynamic range of grayscale value differences, and ultimately, improve the overall contrast of the image [22,23]. The processing method is denoted in Equation 3.


$$P_{f}\left(i\right)=\frac{n_{i}}{N},i=0,1,...,L\text{-1}$$ (3)

In Equation 3, $P_{f}\left(i\right)$ means the frequency of the grayscale image f with a grayscale level of i. n means the amount of pixels, N means the total amount of pixels, and L means the total amount of grayscale levels. The cumulative distribution function is shown in Equation 4.


$$cdf_{f}(i)=\sum_{j=0}^{i}P_{f}\left(i\right)=\sum_{j=0}^{i}\frac{n_{i}}{N}$$ (4)

It is mapped and the grayscale level of the mapping is shown in Equation 5.


$$t=\text{int}[(L-1)cdf_{f}(i)+0.5]$$ (5)

In Equation 5, t represents the grayscale level of the mapping. The final histogram obtained is shown in Equation 6.


$$P_{g}(t)=\frac{n_{t}}{N}$$ (6)

**Fig 3:**
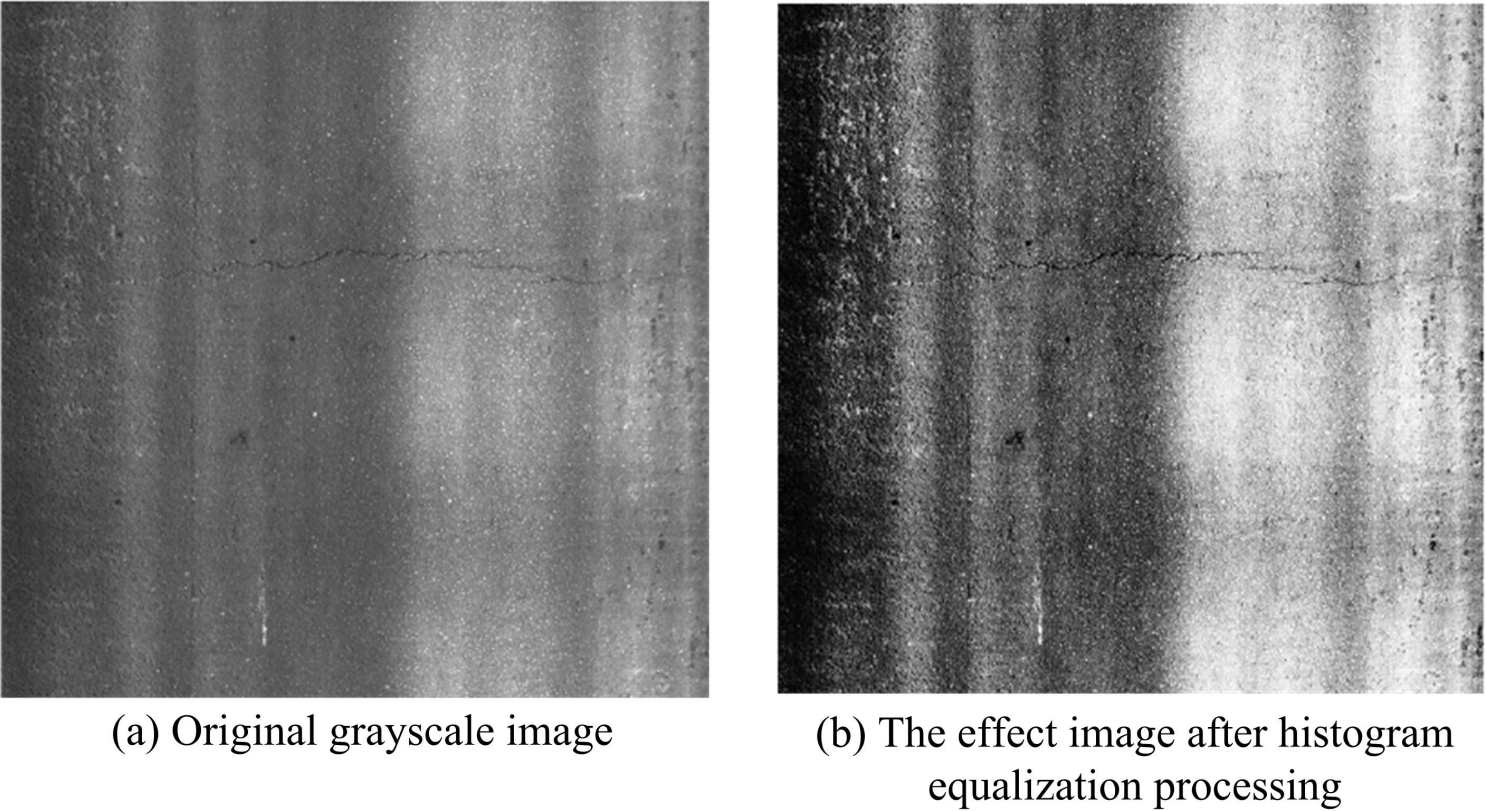
The image effect before and after histogram equalization processing.

In Equation 6, $P_{g}$ represents the updated histogram. The images before and after histogram equalization processing are indicated in Fig 3.

In Fig 3, the transformation of the statistical distribution of pixel values in the image results in a more uniform pixel value distribution in the output image. Fig 3a depicts the original grayscale image, while Fig 3b depicts the influence of road surface cracks following histogram equalization processing. Following the implementation of histogram equalization, a notable enhancement in the contrast and brightness of the image is evident. In practical applications, the presence of noise can impede the accurate extraction of image features. Therefore, it is also necessary to perform noise reduction on images [24,25]. Common image denoising methods for different types of noise in images include median filtering, mean filtering, wavelet transform, etc. [26]. Considering the characteristics of UAV RC images, gradient inverse weighting is selected for denoising processing. In reciprocal gradient weighting, each data point of the signal is assigned a weight, which is calculated based on the reciprocal gradient of that point [27,28]. The larger the reciprocal of the gradient, the smaller the weight, so the noise points in the signal will be weakened, while the real signal points will be preserved. Gradient reciprocal weighting first calculates the reciprocal gradient of each data point in the signal, and then calculates the weight of each data point based on the magnitude of the reciprocal gradient. Finally, it will multiply the weight of each data point by its own value, and then add all the results to obtain the smoothed signal [29–31]. The filtering process using gradient inverse weighting is shown in Equation 7.


$$g(x,y,i,j)=\frac{1}{\left|f(x+i,y+j)-f(x,y)\right|}$$ (7)

In Equation 7, g(x,y,i,j) represents the reciprocal of the pixel gradient of pixel (x,y) with a grayscale value of f(x,y). The normalized weight matrix is shown in Equation 8.


$$W=\left(\begin{array}{ccc} w(x-1,y-1) & w(x-1,y) & w(x-1,y+1)\\ w(x,y-1) & w(x,y) & w(x,y+1)\\ w(x+1,y-1) & w(x+1,y) & w(x+1,y+1) \end{array}\right)$$ (8)

In Equation 8, W represents the central pixel. If W(x,y) is set to 0.5, the weights of the remaining 8 pixels will also be added to 0.5, thus the relationship is shown in Equation 9.


$$w(x+i,y+j)=\frac{1}{2}\frac{g(x,y;i,j)}{\sum_{i}\sum_{j}g(x,y;i,j)}$$ (9)

### 3.2. Road crack segmentation method based on Iotsu algorithm

To raise the efficiency and accuracy of image processing, it is often necessary to segment the image. The most common methods for IS include threshold-based, edge-based, region-based, etc. [32]. A segmentation method based on the Iotsu algorithm is adopted to address the issue of a large proportion of background and a relatively small proportion of target cracks in UAV captured images, which are difficult to extract. The Otsu algorithm is primarily utilized for background segmentation, employing a thresholding approach. The optimal threshold is identified through the maximization of interclass variance, which delineates the background and foreground regions in the image through a grayscale-based classification. This segmentation strategy is associated with the lowest misclassification probability. The Otsu algorithm is denoted in Equation 10.


$$\left\{ \begin{array}{cc} & p1^{*}m1+p2^{*}m2=mG\\ & \text{p1+p2=1} \end{array}\right.$$ (10)

In Equation 10, p1 and p2 represent the probability of pixels being divided into those below the threshold and those above the threshold. m1 and m2 respectively represent the mean values of pixels below the threshold and pixels above the threshold. mGmeans the global mean of the image. The expression for inter class variance is denoted in Equation 11.


$$\sigma^{2}=p1{\left(m1-mG\right)}^{2}+p2{\left(m2-mG\right)}^{2}$$ (11)

In Equation 11, $\sigma^{2}$ represents the overall variance. Equation 11 is simplified and organized to obtain Equation 12.


$$\sigma^{2}=p1p2{\left(m1-m2\right)}^{2}$$ (12)

In Equation 12, the Otsu threshold is the gray level k that maximizes the equation. Among them, p1 calculation is shown in Equation 13.


$$p1=\sum_{i=0}^{k}p_{i}$$ (13)

The calculation of pixel mean m1 and m2 is shown in Equation 14.


$$\left\{ \begin{array}{cc} & m1=1/p1*\sum_{i=0}^{k}ip_{i}\\ & m2=1/p2*\sum_{i=k+1}^{L-1}ip_{i} \end{array}\right.$$ (14)

In Equation 14, L represents that there are L types of grayscale values for pixels. According to the above formula, traverse the gray levels and find the L that maximizes Equation 12, which is the Otsu threshold. However, due to its sensitivity to noise and target size, the inter class variance method only produces good segmentation results for images with a unimodal inter class variance. In the UAV images used in this study, there is a significant disparity in the size ratio between the target cracks and the background, and the inter class variance criterion function may exhibit bimodal or multimodal patterns, resulting in less than ideal performance. In response to the above issues, research has made certain improvements to the Otsu algorithm. The improvement method addresses the threshold selection impact caused by the large difference between the target crack and the background. The probability of pixels with grayscale values as the threshold in the histogram is used as the weight factor, and the pixel distribution probability weight of the threshold grayscale value is replaced with the pixel distribution probability of grayscale values in the histogram threshold domain. The maximum inter class variance after improvement is shown in Equation 15.


$$\sigma^{2}=[1-\nu (k)][P_{1}(k)(m_{1}(k)-m{)}^{2}]+\nu (k)[P_{2}(k)(m_{2}(k)-m{)}^{2}]$$ (15)

**Fig 4:**
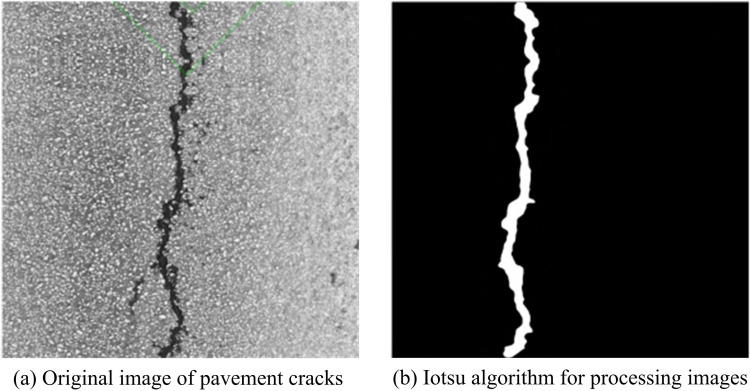
Iotsu algorithm processing effect diagram.

In Equation 15, ν(k) represents the probability of pixel distribution of the threshold within the domain. The effectiveness of the Iotsu algorithm is shown in Fig 4.

In Fig 4, the Iotsu algorithm processed the original UAV captured images to avoid the problem of peak protrusion. Fig 4a showcases the original RC image, and Fig 4b showcases the image processed by the Iotsu algorithm. After modifying the histogram probability weighting factor using the Iotsu algorithm, the image crack targets are well extracted.

### 3.3. Feature extraction and recognition of road cracks based on damage density

**Fig 5:**
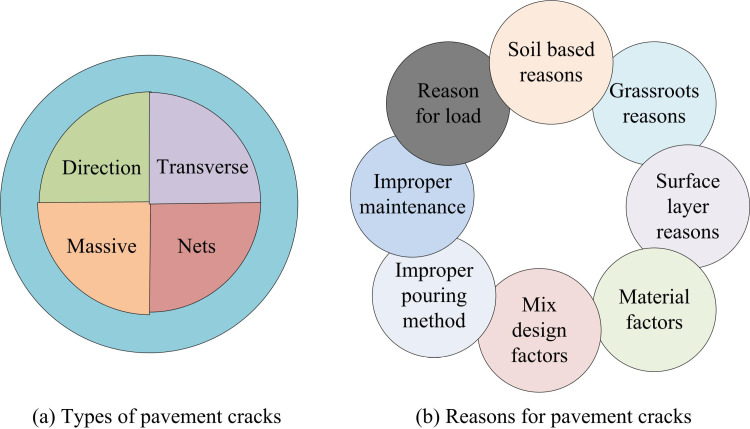
Reasons and types of road cracks.

An RC feature extraction and recognition method based on Dd (Iotsu-Dd) is developed for identifying RCs. Firstly, the causes of road surface cracks are summarized and identified, and then the types of cracks are further distinguished using projection. The causes and types of RCs are shown in Fig 5.

In Fig 5a, the causes of RCs include factors such as materials, climate, load, and improper maintenance. In Fig 5b, the types of cracks are divided into longitudinal, transverse, block shaped, and mesh shaped. According to different types of RCs, the features that need to be extracted are also different. Among them, block and mesh cracks are estimated based on their area, while the other three types of cracks are extracted based on their length and width characteristics. To avoid misclassification issues, a comprehensive consideration is given to the number of crack pixels, the maximum difference in crack projection on the x and y axes. The projection representation of cracks on the x and y axes is shown in Equation 16.


$$\left\{ \begin{array}{cc} & Num(x_{i})=\sum_{j=1}^{M}g(i,j)\\ & Num(y_{i})=\sum_{i=1}^{N}g(i,j) \end{array}\right.$$ (16)

In Equation 16, M and N denote the size of the binary image of RCs. Based on UAV images, pixel size can be easily calculated, making it easier to determine crack length. The width calculation is denoted in Equation 17.


$$D=\frac{S}{L}$$ (17)

In Equation 17, D indicates the mean width of the crack, S indicates the crack area, and L indicates the crack length. The calculation for estimating the crack area is shown in Equation 18.


$$S=\alpha^{2}(x_{\max}-x_{\min})(y_{\max}-y_{\min})$$ (18)

**Fig 6:**
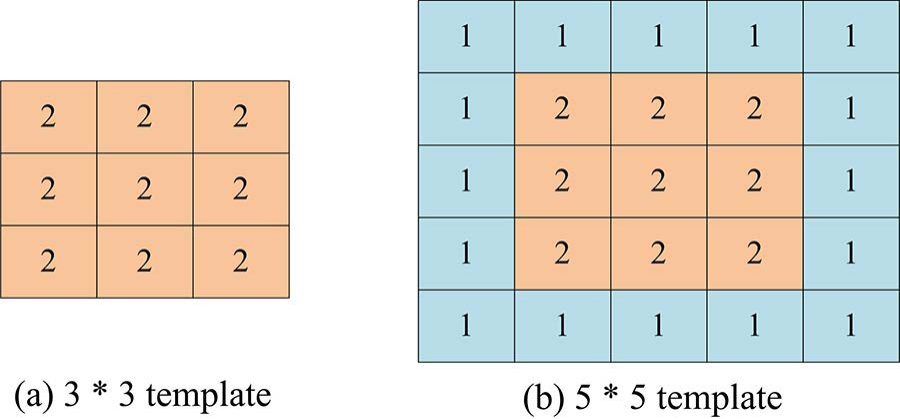
Template for road damage density factor.

In Equation 18, α represents the length value of a single pixel in the road surface. $x_{\max}$, $x_{\min}$ and $y_{\max}$, $y_{\min}$ respectively represent the max and mini values of pixels in the horizontal and the vertical axes. After identifying the types of cracks and analyzing their characteristics, to achieve the recognition of cracks, a feature extraction method grounded on Dd is developed. The method uses a filtering operator with a center of 2. The value is inversely proportional to the distance from the center, with larger values occurring nearer to the center and smaller values occurring farther away, as shown in Fig 6.

Fig 6a shows a 3 * 3 filter operator template, and Fig 6b shows a 5 * 5 filter operator template. Among them, the center position is set to 2. Features are extracted from binary images segmented by the Iotsu algorithm, with the target crack pixel value set to 1 and the background set to 0. If the scan is 1, align it with the center position. If the scan is 0, do not perform any operation. At the same time, convolution operations are performed on the filtering operator and the image, represented by $S_{1}$ and $S_{2}$ respectively, and the resulting values are utilized as the point of pixel value 1. Based on the correlation of RC characteristic values, the type of crack can be determined, as shown in Equation 19.


$$\left\{ \begin{array}{cc} & \lambda_{1}=\frac{\left(S_{1}-\lambda_{0}\right)}{\lambda_{0}}\\ & \lambda_{2}=\frac{\left(S_{2}-\lambda_{0}\right)}{\lambda_{0}} \end{array}\right.$$ (19)

**Fig 7:**
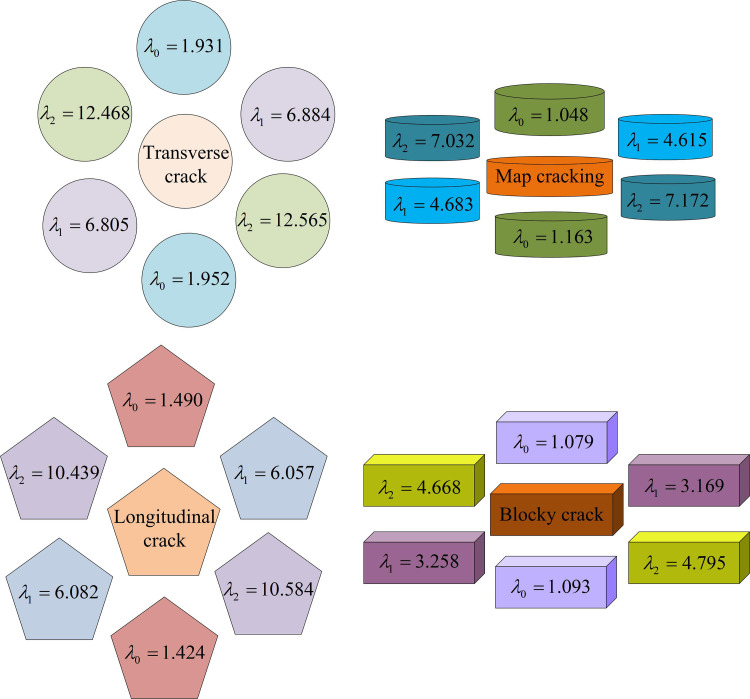
Four types of road crack image feature values.

In Equation 19, $\lambda_{0}$, $\lambda_{1}$, and $\lambda_{2}$ are different eigenvalues. The classification of cracks is determined based on their characteristic values, as shown in Fig 7.

In Fig 7, there are significant discrepancies in the attribute values observed among the four distinct RC images. The $\lambda_{0}$ of transverse cracks is generally stable at around 2, while the $\lambda_{0}$ of longitudinal cracks is between 1.4–1.5. Although there is not much difference in $\lambda_{0}$ characteristic values between block and mesh cracks, there is a notable difference in $\lambda_{1}$ and $\lambda_{2}$ characteristic values. Based on the comparison of feature values, recognition can be performed.

## 4. Analysis of road crack recognition based on uav images and Iotsu algorithm

To validate the efficacy of the developed RC recognition method grounded on UAV images and Iotsu algorithm, the performance of the developed Iotsu algorithm was first validated. Subsequently, common image recognition comparison algorithms and the unimproved Otsu recognition model (Otsu-Dd) were selected for validation of actual road surface cracks.

### 4.1. Performance analysis of image segmentation algorithm based on Iotsu

**Fig 8:**
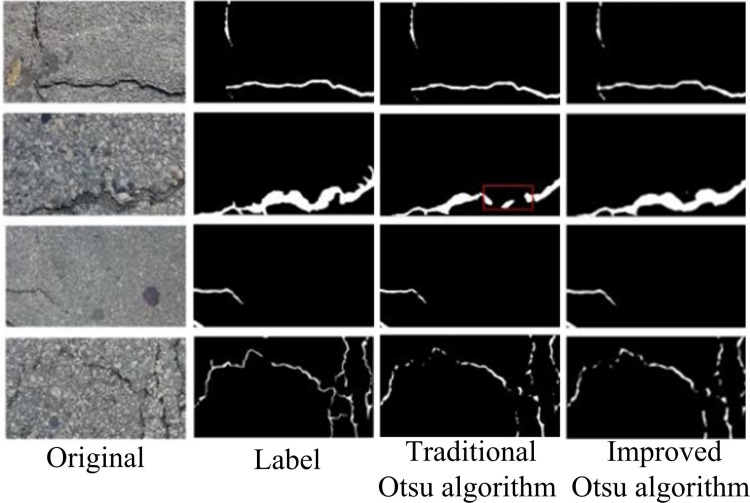
Comparison of image segmentation performance of Otsu algorithm before and after improvement.

To validate the efficacy of the developed RC segmentation method grounded on the Iotsu algorithm, performance tests were carried out on the Iotsu algorithm. The experimental environment was set with a UAV image size of 960 × 540 pixels, an initial learning rate of 0.01, a batch size of 8, an iteration count of 3500, and 100 rounds of training. The images captured by the UAV were preprocessed, including image enhancement and crack type labeling, and ultimately select 2000 images. The dataset used the CrackForest dataset, which is an annotated RC image database, containing over 5000 images of potholes on outdoor roads. Each image was manually reviewed and validated by computer vision experts at Datacluster Labs. A total of 1,000 UAV aerial images were selected for experimentation and randomly divided into two sets: a training set and a testing set, with an 8:2 ratio. The original image, the traditional Otsu threshold segmentation image, and the improved Otsu algorithm segmentation image were contrasted. The comparison results are shown in Fig 8.

**Fig 9:**
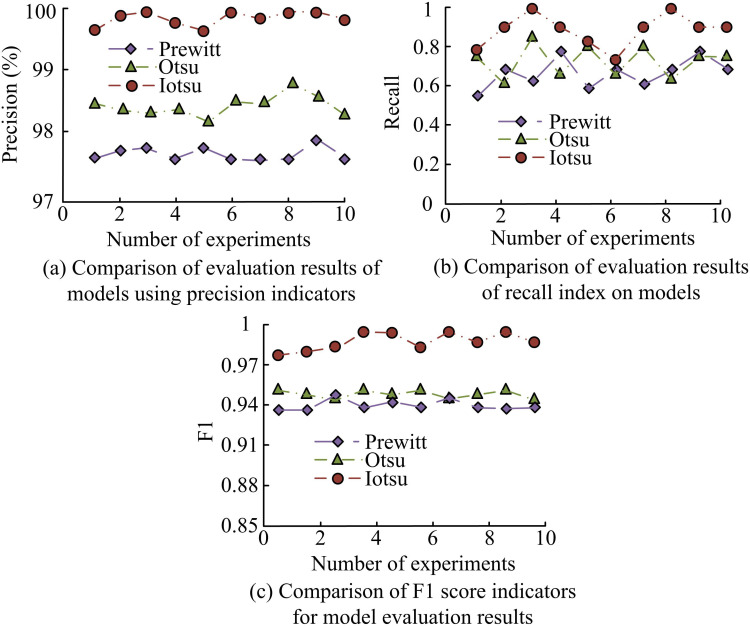
Comparison of precision, recall, and F1 scores of three algorithms.

In Fig 8, the traditional Otsu algorithm suffered from recognition loss in IS at the highlighted positions, and there were certain differences between image recognition and labeling. The Iotsu algorithm had a good segmentation effect on images, and compared with labels, its recognition effect on different types of RCs was almost completely consistent. The outcomes denoted that the Iotsu algorithm had better IS effectiveness compared to the traditional Otsu algorithm and could better adapt to RC IS tasks. Considering that different segmentation algorithms have stronger persuasiveness, the edge detection algorithm Prewitt operator was chosen as the comparative algorithm for IS performance comparison. Precision, Recall, and F1 score were selected for evaluation, and the findings are indicated in Fig 9.

According to Fig 9a, in the Precision comparison, the average Precision of Prewitt algorithm was 97.6%, Otsu algorithm was 98.4%, and Iotsu algorithm was 99.6%. According to Fig 9b, in the Recall evaluation, the average Recall rate of Prewitt algorithm was 0.78, Otsu algorithm was 0.81, and Iotsu algorithm was 0.94. According to Fig 9c, in the F1 score evaluation, the average F1 score of Prewitt algorithm was 0.87, Otsu algorithm was 0.89, and Iotsu algorithm was 0.98. The experimental data shows that the Iotsu algorithm performed the best in all three evaluation metrics, indicating that the Iotsu algorithm had the best performance in IS tasks.

### 4.2. Performance analysis of road crack recognition model based on Iotsu algorithm

**Fig 10:**
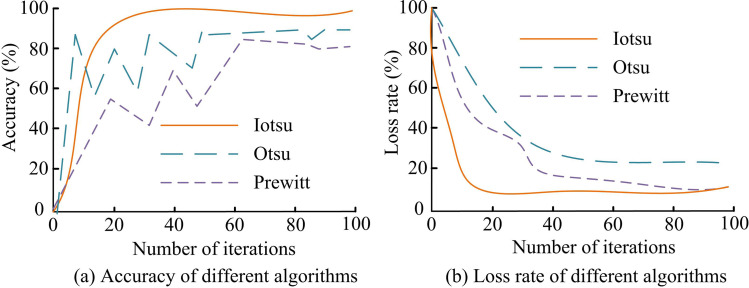
Comparison of training accuracy and loss rate of three models.

To prove the efficacy of the developed recognition method, experiments were conducted from different perspectives to analyze the efficacy of the Iotsu-Dd recognition model. Different models were trained in the training set and analyze the training accuracy and loss rate of each model in the training set. The outcomes are denoted in Fig 10.

**Fig 11:**
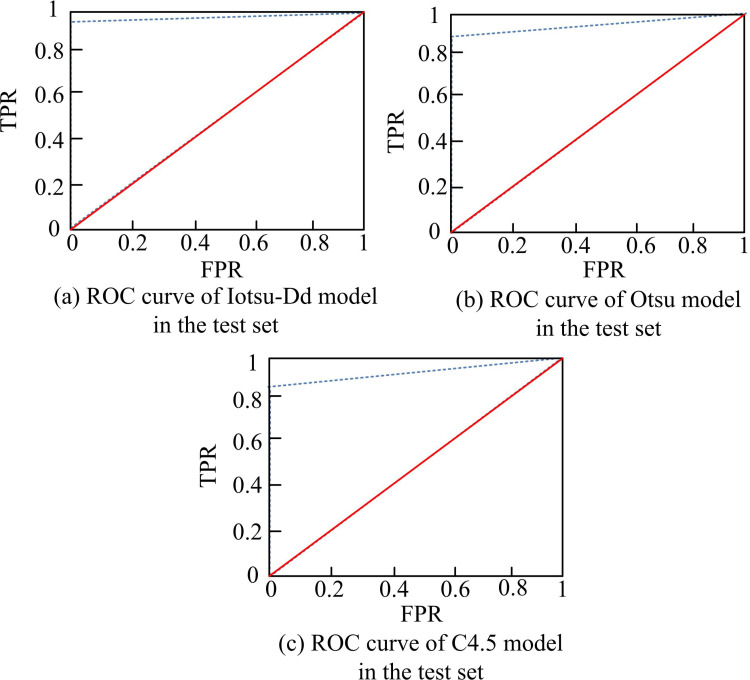
Comparison of ROC curves of three models in the test set.

In Fig 10a, the accuracy of Iotsu-Dd tended to 99% after 18 training sessions. However, otsu-Dd experienced significant fluctuations during the early stages of training and stabilized after 50 iterations, with an accuracy rate ultimately stabilizing at 92.56%. When the Prewitt recognition model was iterated about 65 times, its accuracy approached 90.21%. From Fig 10b, the Iotsu-Dd recognition model had a loss rate approaching 3.41% after about 8 iterations, while Otsu-Dd reached stability after about 40 iterations, with a minimum loss rate of 9.68%. The Prewitt model converged to 5.18% at 36 iterations. Experimental data showed that the developed Iotsu-Dd exhibited higher recognition accuracy and lower loss rate in the training set, and had high stability and convergence efficiency. To validate the recognition efficacy of the model, its ROC curve on the test set was analyzed, as shown in Fig 11.

**Fig 12:**
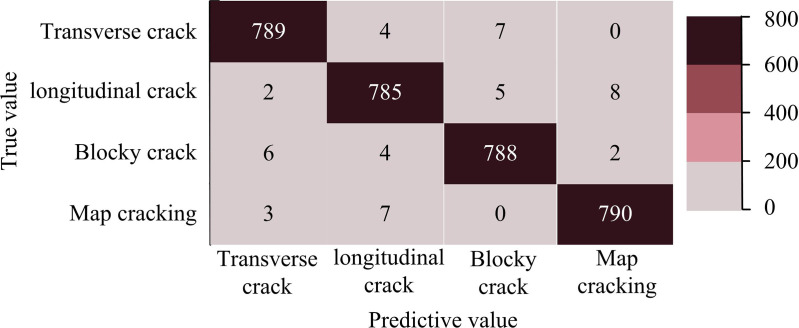
Comparison of ROC curves of three models in the test set.

According to Fig 11a, the Iotsu-Dd model had an AUC value of 0.954 in the test set. The shape formed by the curve was close to the upper left corner, almost forming a right angled triangle. According to Fig 11b, the AUC value of the Otsu-Dd model was 0.882, which was smaller compared to the Iotsu-Dd model. According to Fig 11c, the AUC value of the Prewitt model was the smallest compared to the other two models, at 0.845. The experimental data showed that the Iotsu-Dd model had the highest AUC value among the comparison models, indicating its high recognition accuracy in image crack recognition. The recognition efficacy of the Iotsu-Dd model was analyzed. Its confusion matrix is denoted in Fig 12.

**Fig 13:**
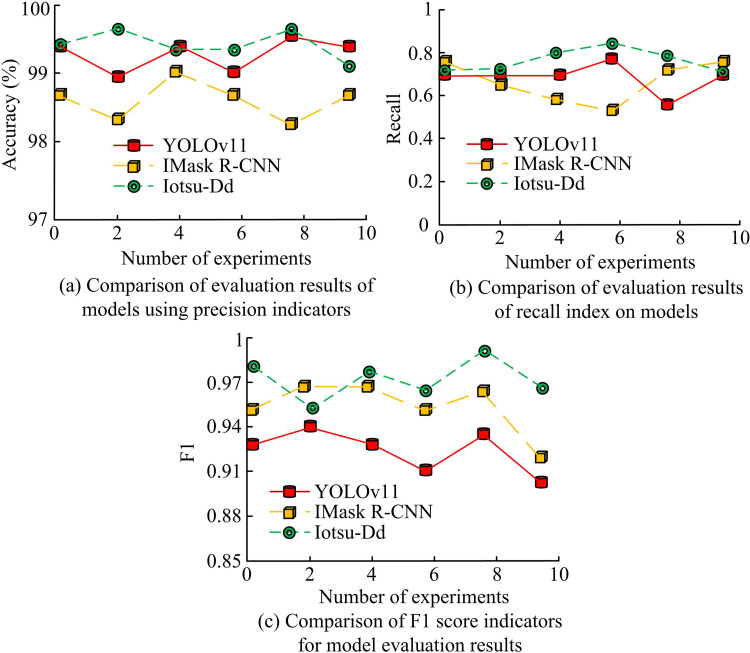
Comparison of precision, recall, and F1 score among different models.

According to Fig 12, the Iotsu-Dd model accurately predicted 789 transverse cracks, 785 longitudinal crack samples, 788 block cracks, and 790 mesh cracks. In the identification of transverse cracks, the amount of misclassified longitudinal cracks was 2, the number of misclassified block cracks was 6, and the number of misclassified mesh cracks was 3. In the identification of longitudinal cracks, there were 4 misclassifications of transverse cracks, 4 misclassifications of block cracks, and 7 misclassifications of network cracks. In the identification of block cracks, there was a misclassification of 7 horizontal cracks and 5 vertical cracks. In the identification of network cracks, the amount of misclassified longitudinal cracks was 8, and the number of misclassified block cracks was 2. The experiment data indicated that the Iotsu-Dd model performed well in recognizing samples in the test set, with high recognition accuracy, indicating that the Iotsu-Dd model had high recognition accuracy. Based on the images collected by the drone, the Iotsu Dd model was validated through examples, and the current advanced models were selected as the comparison models. One is YOLOv11 [33]. The other is an improved Mask Based on Region Convolutional Neural Network (IMask R-CNN) [34]. The study examines the recognition performance of the model using indicators such as model accuracy, recall, and F1 score. The results are shown in Fig 13.

**Fig 14:**
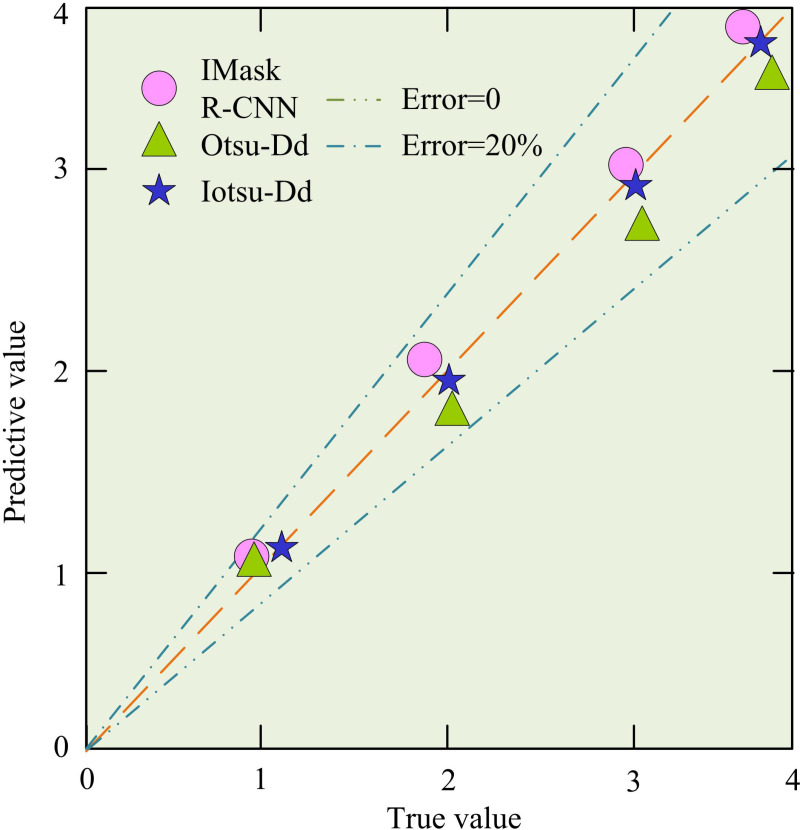
Comparison experimental results of three model errors.

As shown in Fig 13a, the YOLOv11 model has an average accuracy of 99.1% in identifying cracks in drone captured images, the IMask R-CNN has an average accuracy of 98.6%, and the Iotsu Dd recognition model has an average accuracy of 98.2%. According to Fig 13b, the recall rates of the three models are 0.72, 0.76, and 0.75, respectively. According to Fig 13c, the F1 scores of the three models are 0.83, 0.86, and 0.85. Experimental data shows that in the actual image recognition results collected by unmanned aerial vehicles, the recognition performance of the Iotsu Dd model is slightly inferior compared to current advanced image recognition models, but still at a high level. Considering the scarcity of sample data and cost issues in actual detection tasks, the proposed method has the highest cost-effectiveness. To visually analyze the recognition error of the actual image of the model, an error comparison experiment was set up, as shown in Fig 14. 1, 2, 3 and 4 on the axis in the figure represent different types of crack labels in the image.

**Fig 15:**
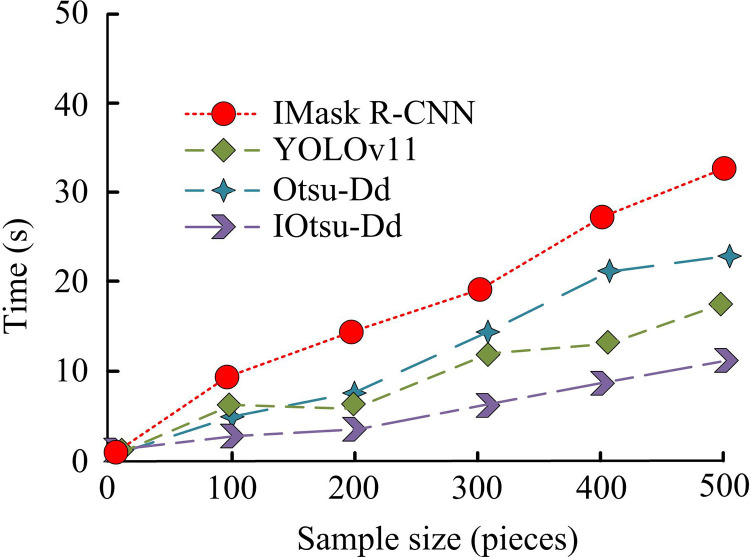
Comparison of the efficiency of UAV image processing using different models.

In Fig 14, the orange dashed line means the error of 0, and the blue dashed line means the percentage error line with an error of 20%. The IMask R-CNN model error results were more inclined towards the percentage error line, with an average error of 11.4%. The Otsu-Dd error results were closer to the orange dashed line compared to SVM, with an average error of 9.4%. The Iotsu-Dd model was almost always on the dotted line with zero error, with an average error of 2.4%. Experimental data indicated that the Iotsu-Dd model had the highest accuracy and strong reliability in identifying images captured by UAVs. To address the efficiency issue of UAV image processing, a comparative experiment was designed for research, as shown in Fig 15.

As shown in Fig 15, the deep learning model IMask R-CNN has low efficiency in drone image processing and takes 32 seconds to complete. The current advanced recognition algorithm YOLOv11 has relatively good processing efficiency, taking 16 seconds to complete the recognition of 500 images. The Otsu DD recognition model took 22 seconds to complete. The IOtsu Dd recognition model proposed by the research institute was completed in 11 seconds. Experimental data shows that the IOtsu Dd recognition model has the highest efficiency in processing drone captured images and can be used for processing drone road crack captured images. The ablation experiment is shown in Table 1.

**Table 1:** Ablation experiment.

Model	Precision (%)	Recall (%)	F1 Score
Otsu-Dd	98.4	0.81	0.89
IOtsu-Dd	99.2	0.88	0.93
IOtsu-Dd+Proposed image processing method	99.6	0.94	0.98

According to Table 1, the performance of the baseline model Otsu DD and the proposed model are continuously improving. The improved Otsu algorithm significantly improved the recall rate and F1 score, indicating that the algorithm has good adaptability and superiority in complex environments. The algorithm combined with the proposed image processing method further improves the accuracy and reliability of crack recognition, indicating the importance of appropriate image processing methods for road crack recognition needs, which can effectively enhance the efficiency and accuracy of highway maintenance.

## Conclusion

With the widespread application of drone technology, the recognition method based on UAV images is becoming increasingly important in RC recognition. Based on this, an RC recognition method with UAV images and Iotsu algorithm was developed. The effectiveness of the developed method was verified through performance validation of the Iotsu algorithm and performance analysis of the RC recognition model based on the Iotsu algorithm. The outcomes denoted that the Iotsu algorithm had a good segmentation effect on images, and compared with labels, its recognition effect on different types of RCs was almost completely consistent. The Precision of the Iotsu algorithm was 98.2%, the Recall rate of the Iotsu algorithm was 0.75, and the F1 score of the Iotsu algorithm was 0.85. When trained 18 times, the Precision of Iotsu-Dd tended to 99%, and when iterated around 8 times, its loss rate tended to 3.41%. The Iotsu-Dd model had an AUC value of 0.954 in the test set, and the graph formed by the curve was close to the upper left corner. The accurately predicted number of transverse cracks was 789, the amount of longitudinal crack samples was 785, the amount of block cracks was 788, and the amount of mesh cracks was 790. The IOtsu-Dd recognition model took 11 seconds to process 500 images. The outcomes denoted that the Iotsu algorithm had better IS effectiveness compared to the traditional Otsu algorithm, could better adapt to RC IS tasks, and had high stability and convergence efficiency. Iotsu-Dd had high recognition accuracy and reliability, and also had high efficiency in processing UAV collected images. It can be used for UAV RC collection image processing. However, there is still a problem in the research due to the insufficient clarity of UAV images in capturing some small cracks, resulting in insufficient accuracy in identifying such cracks. In the future, hardware enhancements such as UAV cameras can be considered to further improve the applicability of the method.

## Supporting information

S1 File Minimal data set. (DOCX)

## References

[1] Ning G. Two-dimensional Otsu multi-threshold image segmentation based on hybrid whale optimization algorithm. Multimed Tools Appl. 2023;82(10):15007–26. doi: 10.1007/s11042-022-14041-1

[2] Al-Rahlawee ATH, Rahebi J. Multilevel thresholding of images with improved Otsu thresholding by black widow optimization algorithm. Multimed Tools Appl. 2021;80(18):28217–43. doi: 10.1007/s11042-021-10860-w

[3] Xiong L, Zhang D, Li K, Zhang L. The extraction algorithm of color disease spot image based on Otsu and watershed. Soft Comput. 2020;24(10):7253–63. doi: 10.1007/s00500-019-04339-y

[4] Young DJN, Koontz MJ, Weeks J. Optimizing aerial imagery collection and processing parameters for drone‐based individual tree mapping in structurally complex conifer forests. Meth Ecol Evol. 2022;13(7):1447–63. doi: 10.1111/2041-210X.13860

[5] Ruan J, Cui H, Huang Y, Li T, Wu C, Zhang K. A review of occluded objects detection in real complex scenarios for autonomous driving. Green Energy Intell Transp. 2023;2(3):100092. doi: 10.1016/j.geits.2023.100092

[6] Liu X, Li J, Ma J, Sun H, Xu Z, Zhang T, Yu H. Deep transfer learning for intelligent vehicle perception: a survey. Green Energy Intell Transp. 2023;2(5):100125. doi: 10.1016/j.geits.2023.100125

[7] Yang P, Song W, Zhao X, Zheng R, Qingge L. An improved Otsu threshold segmentation algorithm. IJCSE. 2020;22(1):146–53. doi: 10.1504/IJCSE.2020.107266

[8] Chen L, Gao J, Lopes AM, Zhang Z, Chu Z, Wu R. Adaptive fractional-order genetic-particle swarm optimization Otsu algorithm for image segmentation. Appl Intell. 2023;53(22):26949–66. doi: 10.1007/s10489-023-04969-8

[9] Huang C, Li X, Wen Y. An Otsu image segmentation based on fruitfly optimization algorithm. AEJ. 2021;60(1):183–8. doi: 10.1016/j.aej.2020.06.054

[10] Bhandari AK, Ghosh A, Kumar IV. A local contrast fusion based 3D Otsu algorithm for multilevel image segmentation. JAS. 2020;7(1):200–13. doi: 10.1109/JAS.2019.1911843

[11] dos Santos RC, Galo M, Carrilho AC, Pessoa GG. The use of Otsu algorithm and multi-temporal airborne LiDAR data to detect building changes in urban space. Appl Geomat. 2021;13(4):499–513. doi: 10.1007/s12518-021-00371-6

[12] Hou Y, Liu S, Cao D, Peng B, Liu Z, Sun W, et al. A deep learning method for pavement crack identification based on limited field images. IEEE Trans Intell Transport Syst. 2022;23(11):22156–65. doi: 10.1109/TITS.2022.3160524

[13] Yang Q, Zhou S. Identification of asphalt pavement transverse cracking based on vehicle vibration signal analysis. Road Mater Pavement Des. 2021;22(8):1783–98. doi: 10.1080/14680629.2020.1714699

[14] Ma D, Fang H, Xue B, Wang F, Msekh A, Chan C. Intelligent detection model based on a fully convolutional neural network for pavement cracks. CMES. 2020;123(3):1267–91. doi: 10.32604/cmes.2020.09122

[15] Tran TS, Tran VP, Lee HJ, Flores JM, Le VP. A two-step sequential automated crack detection and severity classification process for asphalt pavements. Int J Pavement Eng. 2022;23(6):2019–33. doi: 10.1080/10298436.2020.1836561

[16] Li B, Qi Y, Fan J, Liu Y, Liu C. A grid‐based classification and box‐based detection fusion model for asphalt pavement crack. CACAIE. 2023;38(16):2279–99. doi: 10.1111/mice.12962

[17] Du Y, Pan N, Xu Z, Deng F, Shen Y, Kang H. Pavement distress detection and classification based on YOLO network. Int J Pavement Eng, 2021;22(13):1659–72. doi: 10.1080/10298436.2020.1714047

[18] Fan L, Li S, Li Y, Li B, Cao D, Wang F-Y. Pavement cracks coupled with shadows: a new shadow-crack dataset and a shadow-removal-oriented crack detection approach. J Adv Sci. 2023;10(7):1593–607. doi: 10.1109/JAS.2023.123447

[19] Ha J, Kim D, Kim M. Assessing severity of road cracks using deep learning-based segmentation and detection. Transp J Sci. 2022;78(16):17721–35. doi: 10.1007/s11227-022-04560-x

[20] Li B, Wang KCP, Zhang A, Yang E, Wang G. Automatic classification of pavement crack using deep convolutional neural network. Int J Pavement Eng. 2020;21(4):457–63. doi: 10.1080/10298436.2018.1485917

[21] Chu C, Wang L, Xiong H. A review on pavement distress and structural defects detection and quantification technologies using imaging approaches. 2022;9(2):135–50. doi: 10.1016/j.jtte.2021.04.00

[22] Nguyen SD, Tran TS, Tran VP, Lee HJ, Piran MdJ, Le VP. Deep learning-based crack detection: a survey. IJPRT. 2023;16(4):943–67. doi: 10.1007/s42947-022-00172-z

[23] Huang Y, Chen J, Huang D. UFPMP-Det: toward accurate and efficient object detection on drone imagery. AAAI. 2022;36(1):1026–33. doi: 10.1609/aaai.v36i1.19986

[24] Safaei N, Smadi O, Masoud A, Safaei B. An automatic image processing algorithm based on crack pixel density for pavement crack detection and classification. Int J Pavement Res Technol. 2022;15(1):159–72. doi: 10.1007/s42947-021-00006-4

[25] Jana S, Thangam S, Kishore A, Sai Kumar V, Vandana S. Transfer learning based deep convolutional neural network model for pavement crack detection from images. Int J Nonlinear Anal Appl. 2022;13(1):1209–23. https://10.22075/IJNAA.2021.24521.2762

[26] Abbas IH, Ismael MQ. Automated pavement distress detection using image processing techniques. Eng Technol Appl Sci. 2021;11(5):7702–8. doi: 10.48084/etasr.4450

[27] Wu Y, Yang W, Pan J, Chen P. Asphalt pavement crack detection based on multi-scale full convolutional network. JIFS. 2021;40(1):1495–508. doi: 10.3233/JIFS-191105

[28] Hasanvand M, Nooshyar M, Moharamkhani E, Selyari A. Machine learning methodology for identifying vehicles using image processing. AIA. 2023;1(3):170–8. doi: 10.47852/bonviewAIA3202833

[29] Ashraf A, Sophian A, Shafie AA, Gunawan TS, Ismail NN. Machine learning-based pavement crack detection, classification, and characterization: a review. Bulletin EEI. 2023;12(6):3601–19. doi: 10.11591/eei.v12i6.5345

[30] Liu J, Yang X, Lau S, Wang X, Luo S, Lee VCS, et al. Automated pavement crack detection and segmentation based on two‐step convolutional neural network. CACAIE. 2020:35(11):1291–305. doi: 10.1111/mice.12622

[31] Malik YS, Tamoor M, Naseer A, Wali A, Khan A. Applying an adaptive Otsu-based initialization algorithm to optimize active contour models for skin lesion segmentation. J X-Ray Sci Technol. 2022;30(6):1169-84. doi: 10.3233/XST-221245 36093674

[32] Qi F, Xie X, Tang Z, Chen H. Related study based on Otsu watershed algorithm and new squeeze-and-excitation networks for segmentation and level classification of tea buds. NPL. 2021;53(3):2261–75. doi: 10.1007/s11063-021-10501-1

[33] Lui MH, Liu H, Tang Z, Yuan H, Williams D, Lee D, Wang Z, et al. An adaptive YOLO11 framework for the localisation, tracking, and imaging of small aerial targets using a pan–tilt–zoom camera network. Eng. 2024;5(4):3488–516. doi: 10.3390/eng5040182

[34] Sahin ME, Ulutas H, Yuce E, Erkoc MF. Detection and classification of COVID-19 by using Faster R-CNN and Mask R-CNN on CT images. Neural Comput Appl. 2023;35(18):13597–611. doi: 10.1007/s00521-023-08450-y 37213321 PMC10014413

