# Supplementary material for: Pavement crack identification method based on IOtsu-Dd algorithm
Source: PLoS One. 2025 May 14;20(5):e0322662. doi: 10.1371/journal.pone.0322662 (PMC12077713; doi:10.1371/journal.pone.0322662)
Supplement: S1 File — (DOCX) [file pone.0322662.s001.docx]

**The data in Figure 9 (a)**

| Number of experiments | Prewitt Precision (%) | Otsu Precision (%) | Iotsu Precision (%) |
| --- | --- | --- | --- |
| 1 | 97.4 | 98.2 | 99.5 |
| 2 | 97.8 | 98.6 | 99.7 |
| 3 | 97.6 | 98.5 | 99.4 |
| 4 | 97.9 | 98.0 | 99.8 |
| 5 | 97.5 | 98.3 | 99.6 |
| 6 | 97.7 | 98.7 | 99.9 |
| 7 | 97.6 | 98.4 | 99.5 |
| 8 | 97.5 | 98.1 | 99.6 |
| 9 | 97.8 | 98.3 | 99.8 |
| 10 | 97.7 | 98.5 | 99.5 |
| Average value | 97.6 | 98.4 | 99.6 |

**The data in Figure 9 (b)**

| Number of experiments | Prewitt recall | Otsu recall | Iotsu recall |
| --- | --- | --- | --- |
| 1 | 0.75 | 0.79 | 0.93 |
| 2 | 0.80 | 0.82 | 0.95 |
| 3 | 0.77 | 0.80 | 0.94 |
| 4 | 0.78 | 0.81 | 0.92 |
| 5 | 0.76 | 0.83 | 0.94 |
| 6 | 0.79 | 0.81 | 0.96 |
| 7 | 0..78 | 0.80 | 0.93 |
| 8 | 0.75 | 0.82 | 0.94 |
| 9 | 0.79 | 0.80 | 0.95 |
| 10 | 0.80 | 0.81 | 0.92 |
| Average value | 0.78 | 0.81 | 0.94 |

**The data in Figure 9 (b)**

| Number of experiments | Prewitt F1 score | Otsu F1 score | Iotsu F1 score |
| --- | --- | --- | --- |
| 1 | 0.84 | 0.88 | 0.96 |
| 2 | 0.88 | 0.90 | 0.97 |
| 3 | 0.85 | 0.89 | 0.96 |
| 4 | 0.86 | 0.89 | 0.95 |
| 5 | 0.84 | 0.90 | 0.96 |
| 6 | 0.87 | 0.89 | 0.97 |
| 7 | 0.86 | 0.89 | 0.96 |
| 8 | 0.83 | 0.89 | 0.96 |
| 9 | 0.87 | 0.89 | 0.96 |
| 10 | 0.88 | 0.89 | 0.95 |
| Average value | 0.86 | 0.89 | 0.96 |

**The data in Figure 10 (a)**

| Number of iterations | Iotsu Accuracy (%) | Otsu Accuracy (%) | Prewitt Accuracy (%) |
| --- | --- | --- | --- |
| 2 | 68.8 | / | / |
| 6 | 86.91 | / | / |
| 8 | 92.47 | / | / |
| 10 | / | 76.1 | / |
| 12 | 96.74 | / | / |
| 13 | / | / | 72.8 |
| 18 | 99.0 | / | / |
| 20 | 99.0 | 78.4 | / |
| 26 | / | / | 76.3 |
| 30 | / | 79.8 | / |
| 39 | / | / | 83.8 |
| 40 | 99.0 | 81.2 | / |
| 52 | / | / | 78.4 |
| 60 | 99.0 | 92.56 | / |
| 65 | / | / | 90.21 |
| 70 | / | 92.56 | 90.21 |
| 73 | / | / | 90.21 |
| 80 | 99.0 | 92.56 | 90.21 |
| 85 | / | / | 90.21 |
| 90 | / | 92.56 | 90.21 |
| 100 | 99.0 | 92.56 | 90.21 |

**The data in Figure 10 (b)**

| Number of iterations | Iotsu Accuracy (%) | Otsu Accuracy (%) | Prewitt Accuracy (%) |
| --- | --- | --- | --- |
| 2 | 68.8 | / | / |
| 6 | 86.91 | / | / |
| 8 | 92.47 | / | / |
| 10 | / | 76.1 | / |
| 12 | 96.74 | / | / |
| 13 | / | / | 72.8 |
| 18 | 99.0 | / | / |
| 20 | 99.0 | 78.4 | / |
| 26 | / | / | 76.3 |
| 30 | / | 79.8 | / |
| 39 | / | / | 83.8 |
| 40 | 99.0 | 81.2 | / |
| 52 | / | / | 78.4 |
| 60 | 99.0 | 92.56 | / |
| 65 | / | / | 90.21 |
| 70 | / | 92.56 | 90.21 |
| 73 | / | / | 90.21 |
| 80 | 99.0 | 92.56 | 90.21 |
| 85 | / | / | 90.21 |
| 90 | / | 92.56 | 90.21 |
| 100 | 99.0 | 92.56 | 90.21 |

**The data in Figure 11 (a)**

| FPR | TPR | AUC |
| --- | --- | --- |
| 0.2 | 0.921 | 0.954 |
| 0.4 | 0.843 |  |
| 0.6 | 0.762 |  |
| 0.8 | 0.537 |  |
| 0.9 | 0.431 |  |

**The data in Figure 11 (b)**

| FPR | TPR | AUC |
| --- | --- | --- |
| 0.2 | 0.874 | 0.882 |
| 0.4 | 0.762 |  |
| 0.6 | 0.634 |  |
| 0.8 | 0.542 |  |
| 0.9 | 0.319 |  |

**The data in Figure 11 (c)**

| FPR | TPR | AUC |
| --- | --- | --- |
| 0.2 | 0.867 | 0.845 |
| 0.4 | 0.742 |  |
| 0.6 | 0.635 |  |
| 0.8 | 0.524 |  |
| 0.9 | 0.318 |  |

**The data in Figure 12**

| True value | / | Predictive value | | | |
| --- | --- | --- | --- | --- | --- |
|  |  | Transverse Crack | Longitudinal Crack | Blocky Crack | Map Cracking |
|  | Transverse Crack | 789 | 4 | 7 | 0 |
|  | Longitudinal Crack | 2 | 785 | 5 | 8 |
|  | Blocky Crack | 6 | 4 | 788 | 2 |
|  | Map Cracking | 3 | 7 | 0 | 790 |

**The data in Figure 13 (a)**

| Number of experiments | YOLOv11 Accuracy (%) | IMask R-CNN Accuracy (%) | Iotsu-Dd Accuracy (%) |
| --- | --- | --- | --- |
| 2 | 99.3 | 98.5 | 98.0 |
| 4 | 99.1 | 98.7 | 98.2 |
| 6 | 99.2 | 98.6 | 98.1 |
| 8 | 99.0 | 98.5 | 98.3 |
| 10 | 99.0 | 98.6 | 98.1 |
| 12 | 99.1 | 98.6 | 98.3 |

**The data in Figure 13 (b)**

| Number of experiments | YOLOv11 Recall | IMask R-CNN Recall | Iotsu-Dd Recall |
| --- | --- | --- | --- |
| 2 | 0.70 | 0.75 | 0.7 |
| 4 | 0.72 | 0.77 | 0.75 |
| 6 | 0.74 | 0.76 | 0.76 |
| 8 | 0.71 | 0.78 | 0.74 |
| 10 | 0.73 | 0.76 | 0.75 |
| 12 | 0.72 | 0.75 | 0.77 |

**The data in Figure 13 (c)**

| Number of experiments | YOLOv11 F1 score | IMask R-CNN F1 score | Iotsu-Dd F1 score |
| --- | --- | --- | --- |
| 2 | 0.80 | 0.90 | 0.84 |
| 4 | 0.83 | 0.83 | 0.85 |
| 6 | 0.84 | 0.88 | 0.86 |
| 8 | 0.85 | 0.70 | 0.85 |
| 10 | 0.79 | 0.95 | 0.87 |
| 12 | 0.87 | 0.80 | 0.81 |

**The data in Figure 14**

| IMask R-CNN (error) | | | | otsu-Dd (error) | | | | Iotsu-Dd (error) | | | |
| --- | --- | --- | --- | --- | --- | --- | --- | --- | --- | --- | --- |
| Crack 1 | Crack 2 | Crack 3 | Crack 4 | Crack 1 | Crack 2 | Crack 3 | Crack 4 | Crack 1 | Crack 2 | Crack 3 | Crack 4 |
| 12.0 | 11.5 | 10.8 | 11.2 | 9.1 | 9.5 | 10.0 | 9.6 | 2.1 | 2.3 | 2.5 | 2.4 |

**The data in Figure 15**

| Time (s) | Sample size (pieces) | | | | | | | | | | | | | | | | | | | |
| --- | --- | --- | --- | --- | --- | --- | --- | --- | --- | --- | --- | --- | --- | --- | --- | --- | --- | --- | --- | --- |
|  | 100 | | | | 200 | | | | 300 | | | | 400 | | | | 500 | | | |
|  | IMask R-CNN | YOLOv11 | otsu-Dd | Iotsu-Dd | IMask R-CNN | YOLOv11 | otsu-Dd | Iotsu-Dd | IMask R-CNN | YOLOv11 | otsu-Dd | Iotsu-Dd | IMask R-CNN | YOLOv11 | otsu-Dd | Iotsu-Dd | IMask R-CNN | YOLOv11 | otsu-Dd | Iotsu-Dd |
|  | 7.2 | 3.1 | 4.9 | 2.8 | 12.7 | 7.3 | 9.6 | 4.2 | 22.4 | 11.2 | 14.1 | 6.6 | 29.1 | 14.9 | 19.8 | 8.4 | 32.0 | 16.3 | 22.7 | 11.2 |
